# Characterization of two candidate genes, NCoA3 and IRF8, potentially involved in the control of HIV-1 latency

**DOI:** 10.1186/1742-4690-2-73

**Published:** 2005-11-23

**Authors:** Sandie Munier, Delphine Delcroix-Genête, Laëtitia Carthagéna, Audrey Gumez, Uriel Hazan

**Affiliations:** 1 Département des Maladies Infectieuses, Institut Cochin, INSERM U567/CNRS UMR-S 8104/Université Paris 5-René Descartes, 22 rue Méchain, 75014 Paris, France; 2 UFR de Biochimie, Université Paris 7-Denis Diderot, 2 Place Jussieu, 75251 Paris, France

## Abstract

**Background:**

The persistence of latent HIV-1 reservoirs is the principal barrier preventing the eradication of HIV-1 infection in patients by current antiretroviral therapy. It is thus crucial to understand the molecular mechanisms involved in the establishment, maintenance and reactivation of HIV-1 latency. Since chromatin remodeling has been implicated in the transcriptional reactivation of the HIV-1 promoter, we assessed the role of the histone deacetylase inhibitor sodium butyrate (NaB) on two HIV-1 latently infected cell lines (U1 and ACH-2) gene expression.

**Results:**

Analysis of microarrays data led us to select two candidate genes: *NCoA3 *(Nuclear Receptor Coactivator 3), a nuclear receptor coactivator and *IRF8 *(Interferon Regulatory Factor 8), an interferon regulatory factor. *NCoA3 *gene expression is upregulated following NaB treatment of latently infected cells whereas *IRF8 *gene expression is strongly downregulated in the promonocytic cell line following NaB treatment. Their differential expressions were confirmed at the transcriptional and translational levels. Moreover, *NCoA3 *gene expression was also upregulated after treatment of U1 and ACH-2 cells with phorbol myristyl acetate (PMA) but not trichostatin A (TSA) and after treatment with NaB of two others HIV-1 latently infected cell lines (OM10.1 and J1.1). *IRF8 *gene is only expressed in U1 cells and was also downregulated after treatment with PMA or TSA. Functional analyses confirmed that NCoA3 synergizes with Tat to enhance HIV-1 promoter transcription and that IRF8 represses the IRF1-mediated activation through the HIV-1 promoter Interferon-stimulated response element (ISRE).

**Conclusion:**

These results led us to postulate that NCoA3 could be involved in the transcriptional reactivation of the HIV-1 promoter from latency and that IRF8 may contribute to the maintenance of the latent state in the promonocytic cell line. Implication of these factors in the maintenance or reactivation of the viral latency may provide potential new targets to control HIV-1 replication in latent viral reservoirs.

## Background

The use of highly active antiretroviral therapy (HAART) in HIV-1 infected individuals has led to a significant decrease of plasma viremia to undetectable levels and has considerably improved the survival and quality of life of infected individuals (reviewed in [1]). However, the presence of cellular reservoirs that contain latent viruses capable of producing infectious particles after cellular activation lead to a rebound of the viral load after interruption of HAART (reviewed in [2]). The persistence of these latently infected viral reservoirs, despite prolonged HAART treatments, represents a major obstacle to the eradication of HIV-1 in infected patients [3-5]. Therefore, a greater understanding of the molecular mechanisms involved in establishment, maintenance and reactivation of viral latency is essential to expect the reduction of latent HIV-1 reservoirs in infected patients.

Latent HIV-1 infection can exist in many reservoirs, such as macrophages and resting memory CD4^+ ^T cells (reviewed in [6]). At the cellular level, two major forms of HIV-1 latency have been described: pre- and post-integration latency [7]. CD4^+ ^T cells in the post-integration state of latency represent the most stable reservoir for HIV-1 (half-life of 43 months) [8]. Several mechanisms have been proposed to account for the low level of transcription observed during post-integration latency (reviewed in [9]): the inaccessibility of the integrated provirus to the transcriptional machinery, the absence in resting cells of transcription factors involved in HIV-1 gene expression, the presence of transcriptional repressors, and the premature termination of HIV-1 transcription elongation due to the absence of the viral protein Tat and its associated cofactors. Moreover, the chromatin structure appears to be involved in the regulation of HIV-1 gene expression (reviewed in [10]). Indeed, a repressive nucleosome (nuc-1), located immediately downstream of the HIV-1 transcription start site under latency conditions, is disrupted upon transcriptional activation of the HIV-1 promoter in response to Tat, phorbol esters and histone deacetylase (HDAC) inhibitors [11]. Transcriptional activation of the HIV-1 promoter in response to PMA involves the recruitment of SWI/SNF chromatin remodeling complex [12] and cellular proteins with histone acetyltransferase (HAT) activity [13]. Therefore, chromatin remodeling plays a significant role in the transcriptional reactivation of the HIV-1 promoter from latency. Identification of host transcription factors that may regulate chromatin structure is thus critical to understand the molecular mechanisms involved in HIV-1 reactivation.

Gene expression analysis using high-density microarrays have provided a greater understanding of host-pathogen interactions (reviewed in [14]). Previous microarray studies on HIV-1 have described changes in cellular genes transcription in response to HIV-1 protein expression (Nef [15,16], Tat [17,18], gp120 [19] or Vpr [20]) or following acute infection of cell lines [21-24] or Peripheral Blood Mononuclear Cells (PBMC) [25]. DNA microarrays have also been used to characterize gene expression in latently infected resting CD4^+ ^T cells in viremic versus aviremic HIV-1 infected individuals [26]. Recently, global gene expression changes in cell lines latently infected with HIV-1 and induced by PMA for completion of viral replication was described by Krishnan *et al. *[27].

To complete the results obtained by Krishnan *et al.*, we used the same strategy to assess the role of the HDAC inhibitor NaB on HIV-1 latently infected cells gene expression. We performed microarray experiments on two HIV-1 latently infected cell lines (U1 and ACH-2) treated or not with NaB to induce viral reactivation. Analysis of microarrays data led us to select two candidate genes encoding transcription factors: NCoA3 (reviewed in [28]), which expression is upregulated following treatment of latently infected cells with NaB, and IRF8 (reviewed in [29]), which expression is downregulated in treated cells. Differential expression of these genes was confirmed at the transcriptional and translational levels. Moreover, *NCoA3 *gene expression was also upregulated after treatment of U1 and ACH-2 cells with PMA but not TSA and after treatment with NaB of two others latently infected cell lines (OM10.1 and J1.1). *IRF8 *gene is only expressed in U1 cells and was also downregulated after treatment with PMA or TSA. Functional analyses confirmed that NCoA3 synergizes with Tat to enhance HIV-1 promoter transcription and that IRF8 represses the IRF1-mediated activation of the HIV-1 ISRE element. Implication of IRF8 in the maintenance and NCoA3 in the reactivation of the viral latency may thus provide new insights into the control of HIV-1 replication in latent viral reservoirs.

## Results

### Microarray analysis

**Figure 1:**
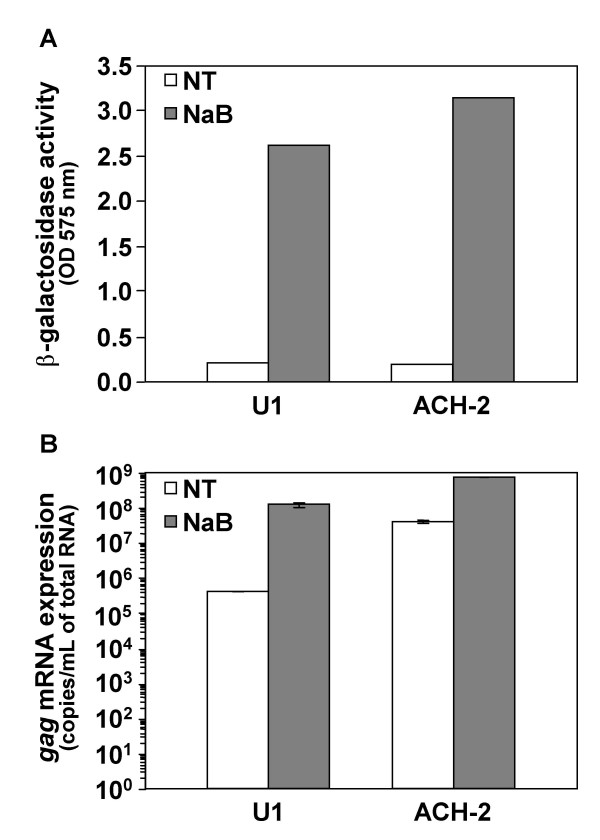
**Analysis of viral reactivation after treatment of U1 and ACH-2 cells with NaB**. U1 and ACH-2 cells were treated or not (NT) with 10 mM of NaB for 24 h and cocultured with P4 indicating cells. β-galactosidase activity was determined after 24 h coculture (A). RNA from U1 and ACH-2 cells treated or not with NaB were extracted after 24 h and *gag *viral mRNA expression was measured by real-time RT-PCR (B). Results are representative of three independent experiments.

In order to understand the molecular mechanisms regulating HIV-1 latency, we studied the modifications of cellular transcription using microarrays in the promonocytic U1 and T CD4^+ ^lymphocytic ACH-2 chronically HIV-1 infected cell lines after reactivation of latency. The two cell lines were treated with 10 mM of the histone deacetylase inhibitor NaB. Viral reactivation was monitored by coculture with P4 indicating cells (Figure 1A) and measuring *gag *viral mRNA expression (Figure 1B). Increase in both β-galactosidase activity and *gag *mRNA expression showed that the viral reactivation after NaB treatment was efficient. Total RNAs were extracted after 24 h and sent to the Affymetrix Microarray Facilities for subsequent hybridization on U-133A microarrays.

The pattern of cellular mRNA from chronically infected cells treated with NaB was compared to that from non-treated cells. We used as specific criteria a log_2 _ratio change ≥ 1 with a change p-value ≤ 0.0001 for increased genes and a log_2 _ratio change ≤ -1 with a 1-change p-value ≥ 0.9999 for decreased genes. Hybridization experiments were performed once. We identified 740 genes that were upregulated by twofold or higher in NaB treated U1 cells and 896 genes that were downregulated, 482 genes in NaB treated ACH-2 cells that had a level increased greater than twofold and 634 genes that had a level decreased greater than twofold (data not shown). Moreover, 260 genes were commonly increased and 337 genes were decreased in both U1 and ACH-2 NaB-treated cells (data not shown). Pathways involved in regulation of transcription, signal transduction, immune response, protein transport, metabolism, apoptosis and RNAs modifications showed altered expression following treatment with NaB. Some of the genes involved in these pathways are shown in Additional Files 1, 2, 3, 4, 5 and 6. Our analysis identified genes that have previously been associated with HIV-1 replication or latency, such as CDK9 [16], Jun [16,23], PSMB10 [27], MAPK1 [26] or OAS1 [30]. This supported the accuracy of our approach, even though, as the hybridization experiments had been performed once, the statistical relevance of the results could not be estimated.

Among the differentially expressed genes, we chose to focus on two candidate genes encoding transcription factors: *NCoA3 *and *IRF8 *(Tables 1 and 2). We selected these two genes based on their biological properties, their described effects on viral replication [31,32] and their differential expression observed by microarray experiments. Indeed, *NCoA3 *and IRF8 gene expression are respectively upregulated and downregulated following treatment with NaB of latently infected cells (Tables 1 and 2). Therefore, NCoA3 and IRF8 could be implicated respectively in the reactivation and maintenance of HIV-1 latency.

**Table 1:** Differential gene expression obtained for NCoA3 and IRF8 mRNAs in U1 cells treated or not with NaB.

Gene	Probe set Name ^*a*^	U1 Signal ^*b*^	U1 Detection p-value ^*c*^	U1NaB Signal	U1NaB Detection p-value	U1NaBvsU1 Signal log_2 _ratio ^*d*^	U1NaBvsU1 Change p-value ^*e*^
*NCoA3*	207700_s_at	17.7	0.01416	98.9	0.000244	2.5	0.000035
	209060_x_at	16.9	0.171387	77.2	0.000244	2.3	0.000023
	209061_at	48.4	0.037598	166.4	0.000732	2.3	0.00002
	209062_x_at	6.3	0.72583	91.8	0.010742	4.5	0.000147
	211352_s_at	7.2	0.303711	68.6	0.00293	3.2	0.000101

*IRF8*	204057_at	707.9	0.000244	47	0.010742	-4	0.99998

^*a *^Affymetrix U133-A reference probe set.

^*b *^Signal intensity of hybridization.

^*c *^Signal detection p-value < 0.048 for specific hybridization.

^*d *^Signal log_2 _ratio > 1 for increased genes and < -1 for decreased genes.

^*e *^Change p-value < 0.0001 for significant increased genes and 1-change p-value > 0.9999 for significant decreased genes.

**Table 2:** Differential gene expression obtained for NCoA3 mRNA in ACH-2 cells treated or not with NaB.

Gene	Probe set Name ^*a*^	ACH-2 Signal ^*b*^	ACH-2 Detection p-value ^*c*^	ACH2NaB Signal	ACH2NaB Detection p-value	ACH2NaBvsACH2 Signal log_2 _ratio ^*d*^	ACH2NaBvsACH2 Change p-value ^*e*^
*NCoA3*	207700_s_at	43.3	0.001953	99.6	0.001221	1.2	0.000241
	209060_x_at	34.5	0.01416	72.9	0.001953	1	0.000273
	209061_at	65.8	0.000732	82.6	0.000732	1.6	0.005409
	209062_x_at	20	0.466064	76.7	0.095215	2	0.000114
	211352_s_at	2.7	0.5	37	0.030273	3.8	0.004481

^*a *^Affymetrix U133-A reference probe set.

^*b *^Signal intensity of hybridization.

^*c *^Signal detection p-value < 0.048 for specific hybridization.

^*d *^Signal log_2 _ratio > 1 for increased genes and < -1 for decreased genes.

^*e *^Change p-value < 0.0001 for significant increased genes and 1-change p-value > 0.9999 for significant decreased genes.

*NCoA3 *gene expression is upregulated following treatment with NaB of both U1 and ACH-2 latently infected cells (Tables 1 and 2). NCoA3 is a nuclear receptor coactivator of the Steroid Receptor Coactivator (SRC) family that interacts with nuclear receptors in a ligand-dependent manner and enhances transcriptional activation *via *histone acetylation and recruitment of general transcription factors and additional cofactors (reviewed in [28]). *NCoA3 *(Unigene Hs. 382168) gene expression in U1 cells is significantly upregulated by 4.9 to 22.6 fold (U1NaBvsU1 signal log_2 _ratio ranging from 2.3 to 4.5 with a change p-value < 0.00015) following treatment with NaB (Table 1). Similarly, *NCoA3 *gene expression is upregulated in NaB-treated compared to non-treated ACH-2 cells by 2 to 13.9 fold but with a lower significance (ACH2NaBvsACH2 signal log_2 _ratio ranging from 1 to 3.8 with a change p-value < 0.0055) (Table 2).

*IRF8 *gene expression is downregulated following treatment of U1 cells with NaB (Table 1). IRF8 is a transcription factor of the Interferon (IFN) Regulatory Factor (IRF) family that binds to IFN-stimulated response element and regulates expression of genes stimulated by IFNs (reviewed in [29]). *IRF8 *(Unigene Hs. 137427) is expressed in the promonocytic cell line U1 (detection signal of 707.9 with a p-value of 0.000244) (Table 1) but is not expressed in the T CD4^+ ^lymphocytic cell line ACH-2 (data not shown). Following NaB treatment, *IRF8 *gene expression in U1 cells is downregulated by 16 fold (U1NaBvsU1 signal log_2 _ratio of -4 with a 1-change p-value of 0.99998) (Table 1).

### Validation of NCoA3 and IRF8 differential transcriptional expression

**Figure 2:**
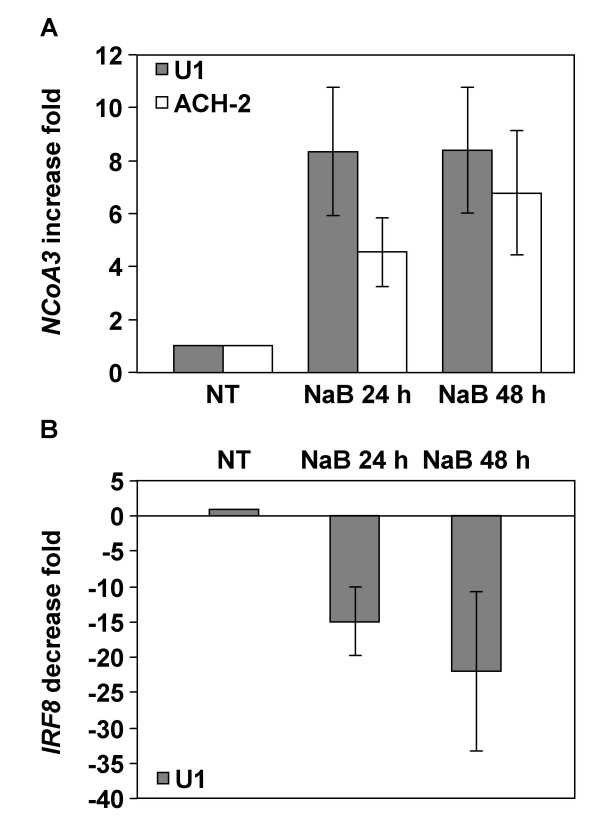
**Real-time RT-PCR analysis of NCoA3 and IRF8 mRNAs expression in NaB-treated U1 and ACH-2 cells**. Total RNAs were isolated from U1 or ACH-2 cells treated or not with NaB for 24 h and 48 h and real-time PCR were performed on cDNAs using gene specific primers for *NCoA3*, *IRF8 *or *Cyclophilin A*. *NCoA3 *and *IRF8 *expressions were normalized to the expression of *Cyclophilin A*. The *NCoA3 *increase fold (A) in U1 (solid bars) or ACH-2 (white bars) cells and the *IRF8 *decrease fold (B) in U1 cells treated with NaB for 24 h and 48 h compared to non-treated (NT) cells were determined. Results represent the means of five independent experiments performed in duplicate.

Real-time RT-PCR quantifications were performed to confirm that *NCoA3 *and *IRF8 *genes were differentially expressed in the NaB-treated chronically infected cells compared to the non-treated cells. We performed quantification on RNA samples obtained from five independent NaB treatments of U1 and ACH-2 cells and real-time RT-PCR experiments were run in duplicate. *NCoA3 *and *IRF8 *expressions were normalized to the expression of *Cyclophilin A*. The results show in Figure 2 represent the *NCoA3 *expression increase fold (Figure 2A) obtained from U1 and ACH-2 cells and the *IRF8 *expression decrease fold (Figure 2B) obtained from U1 cells treated with NaB for 24 h and 48 h compared to non-treated cells. Concerning *NCoA3*, real-time RT-PCR showed an upregulation consistent with microarray data in 24 h NaB-treated U1 cells of 8.34 ± 2.42 fold compared to non-treated cells (Figure 2A). *NCoA3 *gene expression is also increased with a 48 h NaB treatment (upregulation of 8.40 ± 2.33 fold) (Figure 2A). Similarly, an increase of *NCoA3 *gene expression can be observed on ACH-2 cells following treatment with NaB (upregulation of 4.56 ± 1.28 fold in 24 h and 6.80 ± 2.34 fold in 48 h NaB-treated ACH-2 cells) (Figure 2A). Concerning *IRF8*, real-time RT-PCR showed a 14.96 ± 4.85 fold decrease in 24 h NaB-treated U1 cells (Figure 2B) in correlation with the microarray ratio previously obtained. Downregulation of *IRF8 *gene expression is also observed following 48 h NaB-treatment of U1 cells (22.06 ± 11.29 fold decrease) (Figure 2B). Taken together, results from real-time RT-PCR performed on *NCoA3 *and *IRF8 *genes corroborate with those obtained using microarray hybridizations.

**Figure 3:**
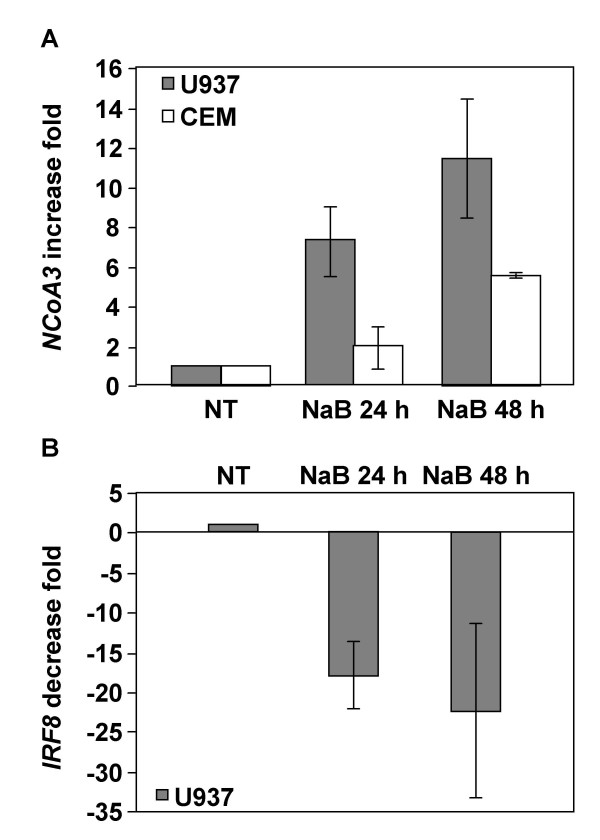
**Real-time RT-PCR analysis of NCoA3 and IRF8 mRNAs expression in NaB-treated U937 and CEM cells**. Total RNAs were isolated from U937 or CEM cells treated or not with NaB for 24 h and 48 h and real-time PCR were performed on cDNAs using gene specific primers for *NCoA3*, *IRF8 *or *Cyclophilin A*. *NCoA3 *and *IRF8 *expressions were normalized to the expression of *Cyclophilin A*. The *NCoA3 *increase fold (A) in U937 (solid bars) or CEM (white bars) cells and the *IRF8 *decrease fold (B) in U937 cells treated with NaB for 24 h and 48 h compared to non-treated (NT) cells were determined. Results represent the means of five independent experiments performed in duplicate.

We next determined whether *NCoA3 *and *IRF8 *gene expression were similarly modified in the uninfected parental cell lines. U937 and CEM cells were subjected to identical treatment and RT-PCR quantifications were performed (Figure 3). NCoA3 is upregulated both in U937 and CEM cells following treatment with NaB (upregulation of 7.32 ± 1.74 fold in 24 h and 11.45 ± 2.95 fold in 48 h NaB-treated U937 cells, upregulation of 1.93 ± 1.04 fold in 24 h and 5.59 ± 0.06 fold in 48 h NaB-treated CEM cells) (Figure 3A). IRF8 is only expressed in the promonocytic cell line U937 and, as in U1 cells, its expression was downregulated after NaB treatment (downregulation of 17.95 ± 4.15 fold in 24 h and 22.32 ± 10.82 fold in 48 h NaB-treated U937 cells) (Figure 3B). Thus, NaB treatment modify *NCoA3 *and *IRF8 *gene expression in uninfected parental cell lines U937 and CEM at a similar level than in chronically infected cells.

**Figure 4:**
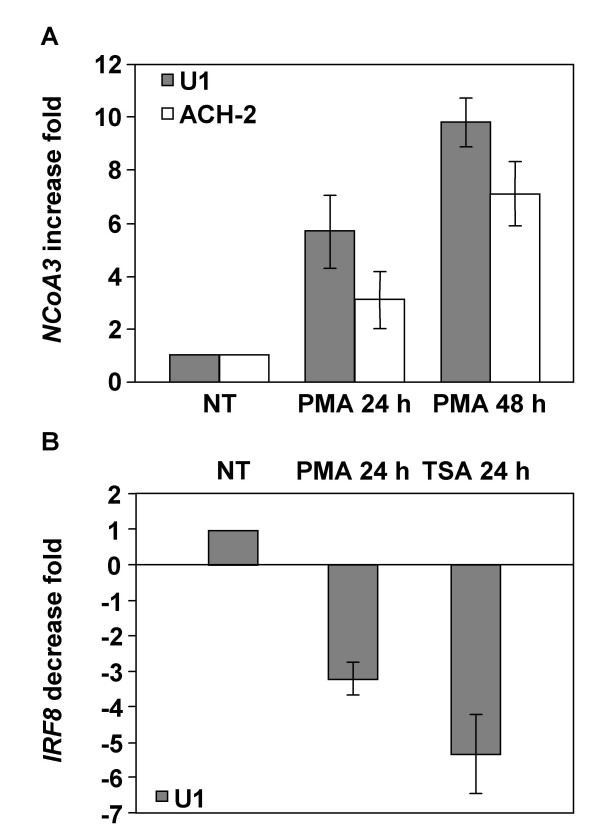
**Real-time RT-PCR analysis of NCoA3 and IRF8 mRNAs expression in PMA- or TSA-treated U1 and ACH-2 cells**. Total RNAs were isolated from U1 or ACH-2 cells treated or not with PMA for 24 h and 48 h or TSA for 24 h and real-time PCR were performed on cDNAs using gene specific primers for *NCoA3*, *IRF8 *or *Cyclophilin A*. *NCoA3 *and *IRF8 *expressions were normalized to the expression of *Cyclophilin A*. The *NCoA3 *increase fold (A) in U1 (solid bars) or ACH-2 (white bars) cells treated with PMA for 24 h and 48 h and the *IRF8 *decrease fold (B) in U1 cells treated with PMA or TSA for 24 h compared to non-treated (NT) cells were determined. Results represent the means of three independent experiments performed in duplicate.

We then performed additional experiments to determine whether the gene expression variations observed could also be mediated by treatments with the phorbol ester PMA and another HDAC inhibitor, TSA. We thus assessed the differential regulation of *NCoA3 *and *IRF8 *gene expression in U1 and ACH-2 cells treated with PMA or TSA (Figure 4). Results indicated that *NCoA3 *expression is upregulated by 24 h and 48 h PMA treatment of U1 and ACH-2 cells (upregulation of 5.70 ± 1.37 fold in 24 h and 9.85 ± 0.90 fold in 48 h PMA-treated U1 cells, upregulation of 3.12 ± 1.05 fold in 24 h and 7.12 ± 1.20 fold in 48 h PMA-treated ACH-2 cells (Figure 4A). However, TSA treatment had no significant effect on *NCoA3 *expression in U1 and ACH-2 cells, although TSA increased viral expression (data not shown). Concerning *IRF8 *expression in U1 cells, PMA and TSA treatments for 24 h induced a decrease of 3.22 ± 0.45 fold and 5.32 ± 1.09 fold, respectively (Figure 4B). These results show that *NCoA3 *expression is upregulated following phorbol ester but not with other HDAC inhibitor treatments in U1 and ACH-2 cells. Moreover, *IRF8 *gene expression in U1 cells is downregulated with PMA or TSA treatments but at a lower extent than with NaB.

**Figure 5:**
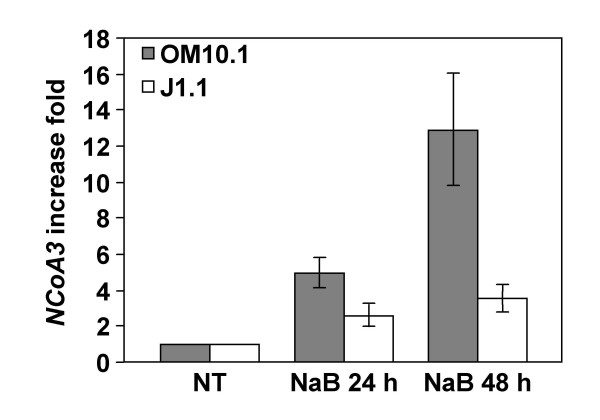
**Real-time RT-PCR analysis of NCoA3 mRNAs expression in OM10.1 and J1.1 cells**. Total RNAs were isolated from OM10.1 or J1.1 cells treated or not with NaB for 24 h and 48 h and real-time PCR were performed on cDNAs using gene specific primers for *NCoA3 *or *Cyclophilin A*. *NCoA3 *expression was normalized to the expression of *Cyclophilin A*. The *NCoA3 *increase fold in OM10.1 (solid bars) or J1.1 cells (white bars) treated with NaB for 24 h and 48 h compared to non-treated (NT) cells were determined. Results represent the means of two independent experiments performed in duplicate.

We also assessed the differential regulation of *NCoA3 *and *IRF8 *gene expression in others chronically HIV-1 infected cell lines. The chronically infected promonocytic OM10.1 and T CD4^+ ^lymphocytic J1.1 cell lines were treated with NaB for 24 h and 48 h and real-time RT-PCR were performed to measure *NCoA3 *and *IRF8 *gene expression. As shown in Figure 5, *NCoA3 *expression is upregulated by 4.94 ± 0.78 fold in OM10.1 and by 2.56 ± 0.64 fold in J1.1 after 24 h NaB treatment. *NCoA3 *expression increased with time of NaB treatment in both cell lines (upregulation of 12.89 ± 3.10 fold in OM10.1 and 3.51 ± 0.69 fold in J1.1 cells) (Figure 5). Like ACH-2 and unlike U1 cells, the T CD4^+ ^lymphocytic J1.1 and the promonocytic OM10.1 cell lines did not express *IRF8 *(data not shown). Thus, the differential regulation of *NCoA3 *but not *IRF8 *gene expression is similar in two other related latently HIV-1 infected cell line models.

### *gag *mRNA activation is correlated with *NCoA3 *mRNA increase and *IRF8 *mRNA decrease

**Figure 6:**
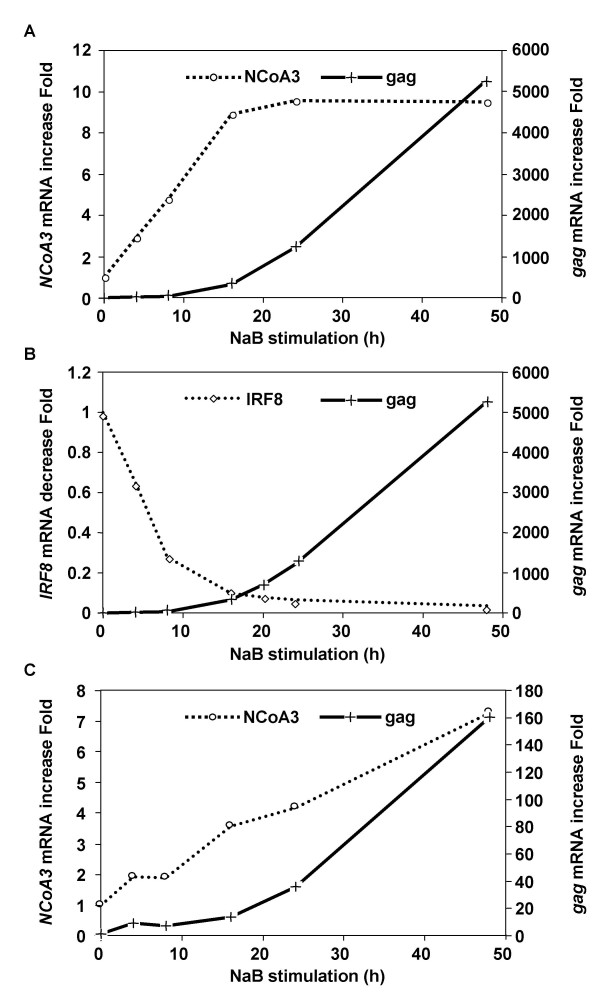
**Analysis of HIV *gag*, *NCoA3*, and *IRF8 *mRNA expression after NaB stimulation on U1 and ACH-2 cells**. U1 (A and B) and ACH-2 (C) cells were stimulated with 10 mM NaB and 5.10^6 ^cells were taken at t = 0, 4, 8, 16, 24, 48 h for RNA extraction to perform qRT-PCR. *NCoA3 *(A and C), *IRF8 *(B) and *gag *(A, B and C) mRNA contents were measured. *Cylophilin A *was used as internal standard. Results represent a representative experiment performed in duplicate.

We performed reactivation experiments at different times, sooner than 24 h and until 48 h. Quantitative RT-PCR experiments were carried out on total RNAs. This was done using U1 cells to analyze both *NCoA3 *mRNA increase (Figure 6A) and *IRF8 *mRNA decrease (Figure 6B) relative to HIV *gag *mRNA along with ACH-2 cells (Figure 6C) to analyze *NCoA3 *mRNA increase relative to HIV *gag *mRNA.

As observed on Figure 6C, the obtained results, both on ACH-2 and U1 cells, clearly show that *gag *mRNA activation occurs after *NCoA3 *mRNA increase and accumulation. Moreover, in U1 cells, *gag *mRNA activation occurs after *IRF8 *mRNA decrease. Shorter kinetics (0 to 8 h) correlated with these results (data not shown).

### Validation of NCoA3 and IRF8 differential translational expression

**Figure 7:**
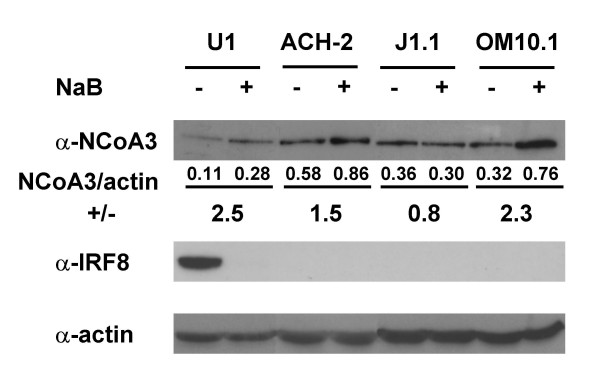
**Western blot analysis of NCoA3 and IRF8 proteins expression**. Nuclear extract (100 μg) from U1, ACH-2, J1.1 and OM10.1 treated (+) or not (-) with NaB for 24 h were resolved by SDS-PAGE and immunoblotted with anti-NCoA3 or anti-IRF8 antibody, as indicated. The amount of protein was normalized using anti-actin antibody. Figures below NCoA3 immunoblot indicated the results of the quantification using Image Tool (Syngene) software of the ratio NCoA3/actin upon NaB-treatment (+) *versus *NCoA3/actin non-treated (-). Results are representative of three independent experiments.

To confirm that the changes seen at the RNA level correlated with protein levels, we performed Western blot experiments on nuclear extract of U1, ACH-2, OM10.1 and J1.1 cells treated or not with NaB for 24 h (Figure 7). Results indicated that NaB increased the expression level of NCoA3 protein in U1, ACH-2, OM10.1 and not in J1.1 cells (Figure 7). Moreover, IRF8 protein expression is strongly downregulated in U1 cells following NaB treatment (Figure 7). These results correlate with the differential expression of *NCoA3 *and *IRF8 *genes observed with both microarray and real-time RT-PCR experiments.

### Transcriptional activation of the HIV-1 promoter by NCoA3

**Figure 8:**
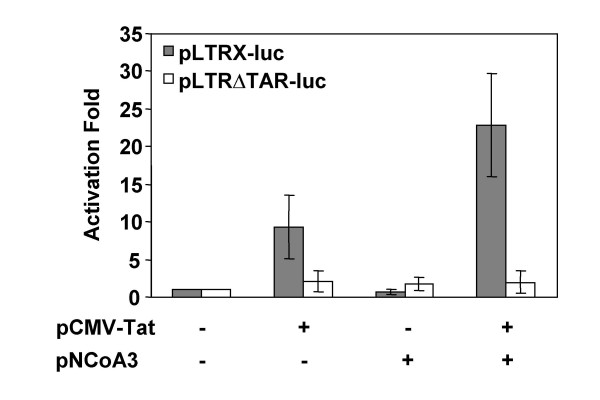
**NCoA3 increases the Tat-stimulated HIV-1 LTR activity**. HEK293 cells were cotransfected with pLTRX-luc (10 ng, grey bars) or pLTRΔTAR-luc (10 ng, white bars) with (+) or without (-) suboptimal amounts of pCMV-Tat (5 ng) and/or pNCoA3 (1 μg) expression vectors. NLI (normalized luciferase index) were measured after 24 h and the activation folds compared to the basal activity of the corresponding pLTR-luc were determined. Results represent the means of five independent experiments.

We analyzed the functional role of NCoA3 on viral transcription by transfection assays. HEK293 cells were cotransfected with pLTRX-luc reporter plasmid containing the luciferase gene under the control of the HIV-1 U3-R promoter region (nt -640 to +78) with or without Tat and/or NCoA3 expression vectors. As shown in Figure 8, NCoA3 increased Tat-stimulated HIV-1 LTR activity by 2.8 ± 1.4 fold. The presence of NCoA3 had synergistic effect on the HIV-1 LTR activity induced by suboptimal expression of Tat. When HEK293 cells were transfected with pLTRΔTAR-luc reporter plasmid lacking the Tat-transactivation response element TAR, Tat was not able to activate the LTR transcription, as expected, and NCoA3 had no effect on the LTR activity (Figure 8). Thus, functional analyses confirm that NCoA3 synergizes with Tat to enhance HIV-1 promoter transcription, as expected [31], and that this effect is dependent on the presence of the TAR region.

### Transcriptional repression of the HIV-1 ISRE element by IRF8

**Figure 9:**
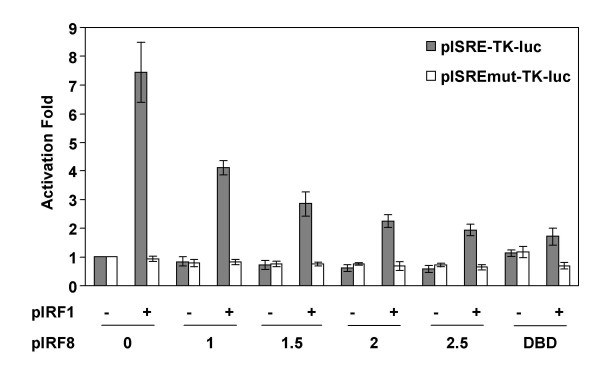
**IRF8 represses the IRF1-mediated activation of the HIV-1 ISRE element**. HEK293 cells were cotransfected with pISRE-TK-luc (250 ng, solid bars) or pISREmut-TK-luc (250 ng, white bars) with (+) or without (-) pIRF1 (250 ng), pIRF8 (1–2.5 μg), or pIRF8-DBD (1 μg) expression vectors. NLI (normalized luciferase index) were measured after 24 h and the activation folds compared to the basal activity of the pISRE-TK-luc or pISREmut-TK-luc were determined. Results represent the means of five independent experiments.

We analyzed the functional role of IRF8 on viral transcription by transfection assays. HEK293 cells were cotransfected with pISRE-TK-luc reporter plasmid corresponding to the HIV-1 IFN-stimulated response element, located downstream transcription start site (nt +194 to +223) [33], with or without IRF1 and/or IRF8 expression vectors. As shown in Figure 9, the basal activity of the ISRE-TK was increased by 7.4 ± 1.0 fold in the presence of IRF1 as expected [32], whereas a decrease was detected in the presence of IRF8 (21.9 ± 10.6 to 41.4 ± 9.5 %). The expression of IRF8 inhibited by 43.5 ± 10.6 to 74.7 ± 2.5 % the IRF1-mediated activation of the ISRE-TK in a dose dependent fashion (Figure 9). The expression of the dominant negative IRF8 DNA-binding domain (IRF8-DBD) inhibited by 76.4 ± 6.5 % the IRF1-mediated activation of the ISRE-TK, as expected [34] (Figure 9). The inhibitory effects of IRF8 and IRF8-DBD expression and activation effect of IRF1 expression was abolished when the ISRE sequence was mutated (pISREmut-TK-luc, Figure 9). These results show that IRF8 represses the ISRE-TK promoter transcription through the ISRE element from the HIV-1 promoter, as expected [32].

## Discussion

The existence of long-lasting HIV-1 reservoirs is the principal barrier preventing the eradication of HIV-1 infection in patients by current antiretroviral therapy. It is thus crucial to understand the molecular mechanisms involved in establishment, maintenance and reactivation of HIV-1 latency. In this study, the role of the HDAC inhibitor NaB on HIV-1 latently infected cells gene expression was explored using microarrays. Since chromatin remodeling is involved in the regulation of HIV-1 gene expression (reviewed in [10]), differential expression of cellular genes in latently infected cells following treatment with NaB might be related to the maintenance and reactivation of latency.

Recently, Krishnan *et al. *[27] described the global gene expression changes in HIV-1 latently infected cell lines treated or not with PMA to induce viral reactivation compared to the uninfected parental cell lines treated under the same conditions. Here, we compared gene expression profiles of two HIV-1 latently infected cell lines (U1 and ACH-2) treated with NaB to that of non-treated corresponding cell lines. We thus avoided identification of genes which differential expression could result from the establishment and cloning of the chronically infected cell lines. Based on our specific criteria, we identified few hundreds of genes affected by NaB treatment implicated in biological pathways previously shown to be modulated by HIV-1 replication. For example, reactivation of latency induced an upregulation of CDK9, the catalytic component of transcription elongation factor b (P-TEFb), which acts in concert with Tat to direct the processivity of HIV-1 transcription. It was shown that CDK9 mRNA and protein levels are induced following T cell activation and Nef expression, and that this correlates with kinase activity, thus enhancing HIV-1 transcription [16,35].

After NaB treatment of latently infected cell lines, we observed an upregulation of genes involved in vesicular transport of protein like syntaxin and nexin. It was found by Chun *et al. *that numerous genes involved in protein/vesicle transport are upregulated in resting T CD4^+ ^cells of viremic patients, strongly suggesting that enhanced activities in secretory pathways may help in the assembly and release of viral particles [26]. Recently, it was shown that multiple genes involved in cholesterol synthesis are induced by Nef [36]. NaB treatment also induced some of these genes (INSIG1, HMGCS1, IDI1, LSS or SREBF1) and could thus enhanced virion infectivity and viral replication.

Krishnan *et al. *have described an increase in expression of several proteasome subunits in ACH-2 cells prior induction of lytic replication by PMA and proposed that the higher expression of proteasomes may lead to increased degradation of HIV-1 mRNA [27]. After induction of lytic replication by NaB, proteasome subunits PSMB10 and PSMB8 were downregulated in ACH-2 and U1 cells, suggesting a role in the maintenance of the latent state. Indeed, reactivation of latency was achieved with proteasome inhibitors [27]. Among the downregulated genes after NaB treatment, we identified genes involved in RNA modifications. Krishnan *et al. *have shown alterations in the expression of DEAD-box and other RNA binding proteins during HIV-1 replication [37]. Especially, DDX18 and DDX39 are upregulated in latently infected cells [37]. After NaB treatment of latently infected cells, we observed a decrease in the expression of these two proteins, thus providing more support for their role in maintaining HIV-1 latency.

The only purpose of our microarray analysis was to identify candidate genes potentially involved in the control of the HIV latency. For this reason, we decided to focus on two candidate genes previously described to influence viral expression and that may be involved in reactivation and maintenance of latency: *NCoA3 *and *IRF8*, respectively. Hybridization experiments were performed once. Consequently, we did not further analyze the statistical relevance of the results and performed complementary approaches to confirm the mRNA variations of the selected candidate genes.

NCoA3 is a nuclear receptor coactivator that enhances ligand-induced transcriptional activation of nuclear receptors (reviewed in [28]). We show that *NCoA3 *(Unigene Hs. 382168) gene expression is upregulated following treatment with NaB of U1 and ACH-2 latently infected cells. This differential transcriptional expression was confirmed by real-time RT-PCR and is also mediated by PMA but not TSA. Upregulation of NCoA3 is thus achieved following phorbol ester but not other HDAC inhibitor treatment. However, NaB and TSA act on different pathways and at different concentrations and target different genes [38]. Transcriptional increase of NCoA3 was observed in parental uninfected corresponding cell lines U937 and CEM and in two others latently HIV-1 infected cell lines, OM10.1 and J1.1. NCoA3 protein level is also upregulated following treatment with NaB in the U1, ACH-2 and OM10.1 cell lines. Moreover, NCoA3 increases the Tat-induced HIV-1 LTR promoter transcriptional activity through the TAR region, in accordance with other data [31]. The differential expression of NCoA3 observed led us to postulate that NCoA3 could be involved in the transcriptional reactivation of the HIV-1 promoter from latency, at low concentrations of Tat.

This hypothesis is supported by several findings. Previous microarray studies on latently infected resting CD4^+ ^T cells in infected individuals have shown an upregulation of NCoA3 gene expression in viremic versus aviremic patients [26]. Moreover, Kino *et al. *showed that NCoA factors improve Tat transactivation of HIV-1 LTR promoter activity and interact with Tat [31]. Tat transactivation activity is mediated by its interaction with components of the basal transcription machinery (including TBP, TAFII250, RNA polymerase II), with kinase complexes able to phosphorylate the C-terminal domain of RNA polymerase II (in particular with the P-TEFb complex composed of cyclin T1/CDK9) and with cellular proteins possessing HAT activity (p300/CBP, P/CAF and GCN5) (reviewed in [39]). Kino *et al. *showed that one member of the family, NCoA2, functions as a Tat coactivator on the HIV-1 LTR by bridging promoter-bound proteins with the Tat-P-TEFb complex through its interaction with Tat and Cyclin T1 [31]. Stimulation of Tat transactivation activity by NCoA3 could involve similar mechanisms.

Furthemore, it has been recently demonstrated that recruitment of HATs to the LTR is an early event in HIV-1 transcriptional activation [13] and that a consequence of histone acetylation is the recruitment of the ATP-dependent chromatin remodeling complex hSWI/SNF to the LTR [12]. NCoA3 could mediate chromatin remodeling by recruitment of additional cofactors with HAT activity (such as p300/CBP and P/CAF) and by an intrinsic HAT activity [40] and may thus contribute to the transcriptional reactivation of the HIV-1 promoter from latency.

IRF8 is a transcription factor that binds to ISRE and regulates expression of genes stimulated by IFNs (reviewed in [29]). IRF8 is able to both activate and repress gene transcription depending on the target gene. We show that *IRF8 *(Unigene Hs. 137427) gene is only expressed in the promonocytic cell line U1 and its expression is strongly downregulated following NaB treatment of these cells. This differential transcriptional expression was confirmed by real-time RT-PCR and is also observed, albeit at lower extent, after PMA and TSA treatments of U1 cells. IRF8 protein level is similarly downregulated following treatment with NaB. Moreover, IRF8 represses the IRF1-mediated activation of the HIV-1 ISRE element of the LTR, in accordance with other data [32]. The decreased expression of IRF8 following reactivation of latency using different molecules suggest that IRF8 may contribute in the maintenance of the latent state in the promonocytic cell line.

It has been shown that binding of specific transcription factors downstream of the HIV-1 transcription start site is crucial to control HIV-1 transcription [33,41]. Among these sites is an ISRE element that recruits IRF1 and IRF2 *in vivo *[33]. Previous studies have investigated the role of IRFs on the modulation of HIV-1 replication (reviewed in [42,43]) and showed that IRF1 activates HIV-1 LTR transcription, interacts with Tat [32] and increases HIV-1 replication [44]. However, IRF8 represses IRF1-Tat-mediated transactivation of the LTR by interfering with IRF1-Tat association [32]. Moreover, it has been shown that IRF8 inhibits HIV-1 replication in T CD4^+ ^lymphocytic and promonocytic cell lines [32,34]. These data support the hypothesis that repression of HIV-1 transcription by IRF8 could be implicated in the maintenance of proviral quiescence in latently infected cells.

Moreover, the result obtained after measurement of *gag*, *NCoA3 *and *IRF8 *mRNA after different times of NaB stimulation clearly showed a correlation between *gag *mRNA increase and *NCoA3 *mRNA increase or *IRF8 *mRNA decrease, respectively. These correlations support the hypothesis that IRF8 and NCoA3 factors may be involved in the control of the HIV latency.

Chronically HIV-1 infected cell lines used in this study provide useful models for studying HIV-1 latency but are not in a quiescent state as cellular reservoirs *in vivo*. Moreover, it has been shown that mutations in the *tat *gene and in the TAR sequence are responsible for the latency observed in U1 and ACH-2 cells, respectively [45,46]. We thus confirmed the differential expression of *NCoA3 *but not *IRF8 g*enes in two others chronically HIV-1 infected cell lines, OM10.1 and J1.1. We will now investigate the involvement of NCoA3 and IRF8 to regulate viral expression in primary cells such as resting T CD4^+ ^lymphocytes or macrophages.

## Conclusion

Additional experiments are currently underway to validate the biological relevance of the differential expression of IRF8 and NCoA3 genes in latency maintenance and reactivation. Since the persistence of integrated HIV-1 genomes despite potent suppression of viral replication is a major obstacle for current antiretroviral therapy, selective disruption of the HIV-1 proviral latency may provide good strategies to decrease latent HIV-1 reservoirs. Thus, identification of cellular genes that are differentially expressed during HIV-1 reactivation of latency is crucial to understand the molecular mechanisms involved in the control of HIV-1 latency.

## Methods

### Cell cultures and treatments

The chronically HIV-1 infected T CD4^+ ^lymphocytic cell lines ACH-2 [47] and J1.1 [48] derived from CEM and Jurkat cells respectively, and the chronically HIV-1 infected promonocytic cell lines U1 [49] and OM10.1 [50] derived from U937 and HL-60 cells respectively, were obtained through the National Institutes of Health (NIH) AIDS Research and Reference Reagent Program. Suspension cell lines were grown in RPMI 1640 (Invitrogen) with 10% fetal bovine serum (Invitrogen), 50 U/mL penicillin, 50 μg/mL streptomycin (Invitrogen) and 2 mM glutamine (Invitrogen). Cells were treated with 10 mM of sodium butyrate (NaB; Sigma), or with 10 ng/mL of PMA (Sigma), or with 300 nM of TSA (Sigma). Cells were harvested generally 24 h and 48 h after treatment and cell viability was estimated before subsequent RNA extraction or nuclear extract preparation. P4 indicator cells are HeLa CD4^+ ^cells carrying the *lacZ *gene under the control of the HIV-1 LTR. P4 and HEK293 cells were grown in DMEM (Invitrogen) containing 5% fetal bovine serum (Invitrogen), 50 U/mL penicillin, 50 μg/mL streptomycin (Invitrogen) and 2 mM glutamine (Invitrogen).

### Plasmids

The pLTRX-luc construct contains the luciferase (luc) gene downstream of the HIV-1 BRU U3-R promoter region (nt -640 to +78) [51]. The pLTRΔTAR-luc construct corresponds to the pLTRX-luc plasmid in which the TAR region (nt +38 to +78) was deleted [51]. The pCMV-Tat expression vector was kindly provided by S. Emiliani (Institut Cochin, Paris, France). The pIRF8 expression vector (pcDNAmycHis-ICSBP) and dominant negative construct pIRF8-DBD, which contains the DNA binding domain of IRF8, were a kind gift of B.Z. Levi (Technion-Israel Institute of Technology, Haifa, Israel). The pNCoA3 expression vector (pcDNA3.1-AIB1) was a kind gift of P.S. Meltzer (NIH, Bethesda, USA) [52]. The pIRF1 construct was generated by cloning the fragment excised from pHuIRF-3-1 (a kind gift of T. Taniguchi, University of Tokyo, Tokyo, Japan) by *Hind*III/*Not*I digestion in the pcDNA3.1 plasmid (Invitrogen). The pISRE-TK-luc and pISREmut-TK-luc constructs were generated by cloning a wild-type (AGGGACTTGAAAGCGAAAGGGAAACCAGAG) or mutated (AGGGACTTGCCCGCGCCCGGGAAACCAGAG) synthetic oligonucleotide corresponding to the HIV-1 BRU ISRE sequence (nt +194 to +223) [33,53] in the pTK-luc plasmid in which the luciferase gene is under the control of the truncated HSV-1 thymidine kinase promoter minimum region [51]. The pCMV-LacZ was kindly provided by M. Alizon (Institut Cochin, Paris, France).

### Total RNA extraction

Total RNAs were extracted using the RNeasy Mini Kit (Qiagen). The procedure included an "on-column" DNase I digestion step according to the manufacturer's instructions. RNA quality was assessed using the Agilent Bioanalyzer 2100 and spectrophotometric analysis prior to cDNA synthesis.

### Microarray experiments

Microarray experiments were performed using the U133-A microarrays (Affymetrix) containing 22283 oligonucleotides spots. Total RNAs obtained from chronically infected U1 and ACH-2 stimulated or not with NaB for 24 h were sent to Dr. C. Thibault (Affymetrix Microarray Facilities, IGBMC, Strasbourg, France) for amplification, labeling and hybridization. Hybridization experiments were performed once. Results were then analyzed with the Mas5.0 Software (Affymetrix) and interpreted using the Data Mining Tool (Affymetrix) and Microsoft Excel softwares. For individual analyses, the p-value cut off was 0.048 as suggested by Affymetrix. For comparative analyses, a log_2 _ratio change ≥ 1 for increased genes and ≤ -1 for decreased genes were defined. Gene expression changes were considered to be significant when the change p-value was ≤ 0.0001 for increased genes and 1-change p-value ≥ 0.9999 for decreased genes.

### Real-time RT-PCR

Quantifications of cellular RNAs were performed using a Light Cycler instrument (Roche Diagnostics). Briefly, cDNAs were synthesized from 1 μg of total RNA with MoMLV reverse transcriptase (Superscript II, Invitrogen) and 1/10^th ^aliquots of the corresponding samples were used for real-time PCR in a 20 μL reaction mixture containing 1X LightCycler FastStart DNA Master SYBR Green I (Roche Diagnostics), 4 mM MgCl_2_, and 500 nM of each primer. The reactions were carried out in duplicate and the results were normalized to the expression of Cyclophilin A. Primers for quantitative PCR were designed using Oligo 6 software. All primer pairs produced single amplification product as determined by melting curve analyses. The sequences of the primers used were (5' to 3'): NCoA3 forward CTTTGGGCATTCCTGAACTTGTC, NCoA3 reverse GCCTCATCACCGCAGCAC, IRF8 forward GGAGTGCGGTCGCTCTGAAA, IRF8 reverse GTCGTAGGTGGTGTACCCCGTCA, Cyclophilin A forward AGTGGTTGGATGGCAAGC, Cyclophilin A reverse GATTCTAGGATACTGCGAGCAAA. PCR reactions were carried out with a denaturation step of 10 min at 95°C followed by forty-five cycles of 10 s at 95°C, 5 s at annealing temperature (55°C for NCoA3 and Cyclophilin A, 59°C for IRF8) and 20 s amplification at 72°C. Quantifications of cDNAs were determined in reference to a standard curve prepared by amplification of serial dilutions of PCR product containing matching sequences. Analyses were performed using the second-derivative-maximum method provided by the Light Cycler quantification software, version 3.5 (Roche Diagnostics).

Quantification of *gag *viral mRNA was performed by real-time RT-PCR as described in [54].

### Nuclear extracts preparation

For nuclear extract preparation, 10.10^6 ^cells were harvested, washed and nuclei were isolated by addition of 150 μL of buffer I (50 mM Tris pH 7.9, 10 mM KCl, 10% glycerol, 1 mM EDTA, 0.2% NP40) followed by a centrifugation at 3000 g for 3 min. Nuclear extracts were prepared by addition of 15 μL of buffer II (20 mM Hepes pH 7.9, 400 mM NaCl, 10 mM KCl, 20% glycerol, 1 mM EDTA) for 20 min at 4°C followed by a centrifugation at 15000 g for 10 min. Protein concentrations were determined by the Bio-Rad protein assay.

### Western blot analysis

Nuclear extracts (100 μg) were loaded on 8% SDS-polyacrylamide gel and the proteins were transferred to nitrocellulose membrane (Hybond-C, Amersham) that was subsequently blocked for 1 h with 5% non-fat dry milk in PBS-T (PBS, 0.05% Tween20) and incubated with antibodies directed against NCoA3 (goat polyclonal anti-ACTR C-20, Santa Cruz Biotechnology, Inc.), IRF8 (goat polyclonal anti-ICSBP C-19, Santa Cruz Biotechnology, Inc.) or actin (mouse monoclonal anti-actin, Calbiochem) for 2 h. The membranes were then washed and incubated with secondary antibodies conjugated to horseradish peroxidase (HRP conjugated rabbit anti-goat (DakoCytomation) or goat anti-mouse (Calbiochem) immunoglobulins). Hybridizations were revealed using an ECL enhanced chemiluminescence kit (ECL, Amersham). The quantification was done using the Image Tools (Syngene) software.

### Transient transfection and enzymatic assays

HEK293 cells were transfected using calcium phosphate co-precipitation method. Cells were lysed 24 h after transfection with a buffer containing 60 mM Na_2_HPO_4_, 40 mM NaH_2_PO_4_, 10 mM KCl, 10 mM MgSO_4_, 2.5 mM EDTA, 50 mM β-mercaptoethanol and 0.125% Nonidet P-40. Luciferase activities were measured as previously described [55]. Cotransfection with pCMV-LacZ plasmid was performed to normalize transfection efficiency and β-galactosidase activities were determined using Chlorophenol red β-D-galactopyranoside (CPRG, Roche Diagnostics) assay as previously described [55]. The normalized luciferase index (NLI) was defined as the ratio of luciferase to β-galactosidase activities.

## Competing interests

The author(s) declare that they have no competing interests.

## Authors' contributions

SM performed the microarray analyses, real-time RT-PCR and drafted the manuscript. DD and LC carried out real-time RT-PCR, Western blot and transfection experiments. AG participated in transfection experiments. UH conceived the study, participated in its design and coordination and helped to draft and finalize the manuscript. All authors read and approved the final manuscript.

## Supplementary Material

Additional File 1 Genes upregulated in U1 and ACH-2 cells. Click here for file

Additional File 2 Genes specifically upregulated in U1 cells. Click here for file

Additional File 3 Genes specifically upregulated in ACH-2 cells. Click here for file

Additional File 4 Genes downregulated in U1 and ACH-2 cells. Click here for file

Additional File 5 Genes specifically downregulated in U1 cells. Click here for file

Additional File 6 Genes specifically downregulated in ACH-2 cells. Click here for file
