# Supplementary material for: Characterization of two candidate genes, NCoA3 and IRF8, potentially involved in the control of HIV-1 latency
Source: Retrovirology. 2005 Nov 23;2:73. doi: 10.1186/1742-4690-2-73 (PMC1310520; doi:10.1186/1742-4690-2-73)
Supplement: Additional File 1 — Genes upregulated in U1 and ACH-2 cells. [file 1742-4690-2-73-S1.doc]

| **Symbol** | **Name** | **U1NaBvsU1 Signal log_2_ ratio** | **ACH2NaBvsACH2 Signal log_2_ ratio** |
| --- | --- | --- | --- |
|  |  |  |  |
| **Transcription** | |  |  |
| HIS1 | HMBA-inducible | 2.7 | 1.1 |
| EPAS1 | endothelial PAS domain protein 1 | 2.4 | 3.1 |
| MYBL1 | V-myb myeloblastosis viral oncogene homolog (avian)-like 1 | 2.4 | 1.7 |
| ZNF277 | zinc finger protein (C2H2 type) 277 | 2.2 | 1.9 |
| STAT3 | signal transducer and activator of transcription 3 | 2.1 | 1 |
| RYBP | RING1 and YY1 binding protein | 2 | 1.5 |
| NFIL3 | nuclear factor, interleukin 3 regulated | 2 | 1.1 |
| YPEL5 | yippee-like 5 (Drosophila) | 1.9 | 2.3 |
| CTNNBIP1 | catenin, beta interacting protein 1 | 1.6 | 2.1 |
| SIRT2 | sirtuin (silent mating type information regulation 2 homolog) 2 | 1.6 | 1.5 |
| TCEA2 | transcription elongation factor A (SII), 2 | 1.6 | 1.5 |
| HDAC3 | histone deacetylase 3 | 1.6 | 1.3 |
|  |  |  |  |
| **Signal Transduction** | |  |  |
| RALGPS1 | Ral GEF with PH domain and SH3 binding motif 1 | 3.9 | 2.1 |
| RRAS | related RAS viral (r-ras) oncogene homolog | 3.3 | 5 |
| IQGAP1 | IQ motif containing GTPase activating protein 1 | 2.8 | 1.2 |
| TNFSF9 | tumor necrosis factor (ligand) superfamily, member 9 | 2.5 | 1.9 |
| GRN | granulin | 2.4 | 2.1 |
| RAB40B | RAB40B, member RAS oncogene family | 2.2 | 1.2 |
| RTN2 | reticulon 2 | 1.8 | 2.3 |
| GPSM2 | G-protein signalling modulator 2 (AGS3-like, C. elegans) | 1.8 | 1.7 |
| ARL3 | ADP-ribosylation factor-like 3 | 1.8 | 1.6 |
| RABL4 | RAB, member of RAS oncogene family-like 4 | 1.8 | 1.3 |
| RPS6KC1 | ribosomal protein S6 kinase, 52kDa, polypeptide 1 | 1.8 | 1.1 |
| PPP2R5B | protein phosphatase 2, regulatory subunit B (B56), beta isoform | 1.7 | 1.8 |
|  |  |  |  |
| **Immune Response** | |  |  |
| SPP1 | secreted phosphoprotein 1 (osteopontin) | 4.1 | 2.9 |
| IFI30 | interferon, gamma-inducible protein 30 | 4 | 2 |
|  |  |  |  |
| **Protein Transport** | |  |  |
| NPC1 | Niemann-Pick disease, type C1 | 2.3 | 3.9 |
| SNAP29 | synaptosomal-associated protein, 29kDa | 1.8 | 1.3 |
| GABARAPL | GABA(A) receptor-associated protein-like 2 | 1.7 | 1.2 |
| SNAP23 | synaptosomal-associated protein, 23kDa | 1.7 | 1.2 |
| GDI1 | GDP dissociation inhibitor 1 | 1.6 | 1.3 |
| STX12 | syntaxin 12 | 1.4 | 1.3 |
| RNP24 | coated vesicle membrane protein | 1.4 | 1 |
|  |  |  |  |
| **Miscellaneous** | |  |  |
| ENPP2 | ectonucleotide pyrophosphatase/phosphodiesterase 2 | 8.7 | 2.1 |
| COL1A1 | collagen, type I, alpha 1 | 6.3 | 3.9 |
| SORBS1 | sorbin and SH3 domain containing 1 | 5.5 | 5.4 |
| TXNIP | thioredoxin interacting protein | 4.5 | 3.3 |
| SAT | spermidine/spermine N1-acetyltransferase | 4 | 2.3 |
| SGK | serum/glucocorticoid regulated kinase | 3.9 | 4.6 |
| HHLA3 | HERV-H LTR-associating 3 | 3.5 | 1.4 |
| SYT11 | synaptotagmin XI | 3.4 | 2.5 |
| CDKN1A | cyclin-dependent kinase inhibitor 1A (p21, Cip1) | 3.3 | 4.9 |
