# Supplementary material for: Characterization of two candidate genes, NCoA3 and IRF8, potentially involved in the control of HIV-1 latency
Source: Retrovirology. 2005 Nov 23;2:73. doi: 10.1186/1742-4690-2-73 (PMC1310520; doi:10.1186/1742-4690-2-73)
Supplement: Additional File 2 — Genes specifically upregulated in U1 cells. [file 1742-4690-2-73-S2.doc]

| **Symbol** | **Name** | **U1NaBvsU1 Signal log_2_ ratio** |
| --- | --- | --- |
|  |  |  |
| **Transcription** | |  |
| KLF2 | Kruppel-like factor 2 (lung) | 3.7 |
| FOS | v-fos FBJ murine osteosarcoma viral oncogene homolog | 3.2 |
| NR4A2 | nuclear receptor subfamily 4, group A, member 2 | 3.2 |
| **NCOA3** | **nuclear receptor coactivator 3** | **2.5** |
| NR4A1 | nuclear receptor subfamily 4, group A, member 1 | 2.4 |
| SSBP2 | single-stranded DNA binding protein 2 | 2.4 |
| MLLT3 | myeloid/lymphoid or mixed-lineage leukemia ; translocated to, 3 | 2.3 |
| SIRT4 | sirtuin (silent mating type information regulation 2 homolog) 4 (S. cerevisiae) | 2.2 |
| USP49 | ubiquitin specific protease 49 | 2.2 |
| BCL6 | B-cell CLL/lymphoma 6 (zinc finger protein 51) | 2.1 |
| SMARCD3 | SWI/SNF related, matrix associated, actin dependent regulator of chromatin | 2.1 |
| ZNF297B | zinc finger protein 297B | 2.1 |
|  |  |  |
| **Signal Transduction** | |  |
| RGS2 | regulator of G-protein signalling 2, 24kDa | 3.9 |
| ANXA1 | annexin A1 | 3.1 |
| LY96 | lymphocyte antigen 96 | 2.9 |
| CHP | calcium binding protein P22 | 2.7 |
| CD69 | CD69 antigen (p60, early T-cell activation antigen) | 2.5 |
| TNFAIP6 | tumor necrosis factor, alpha-induced protein 6 | 2.5 |
| GRK5 | G protein-coupled receptor kinase 5 | 2.3 |
| RGL1 | ral guanine nucleotide dissociation stimulator-like 1 | 2.3 |
| OPTN | optineurin | 2.1 |
| RAB8B | RAB8B, member RAS oncogene family | 2 |
| GNB5 | guanine nucleotide binding protein (G protein), beta 5 | 1.7 |
| TRIM23 | tripartite motif-containing 23 | 1.7 |
| RAB3D | RAB3D, member RAS oncogene family | 1.6 |
|  |  |  |
| **Immune Response** | |  |
| C3 | complement component 3 | 5.4 |
| G1P3 | interferon, alpha-inducible protein (clone IFI-6-16) | 3.1 |
| FTH1 | ferritin, heavy polypeptide 1 | 3 |
| OASL | 2'-5'-oligoadenylate synthetase-like | 2.4 |
|  |  |  |
| **Protein Transport** | |  |
| COPA | coatomer protein complex, subunit alpha | 2.3 |
| ACBD3 | acyl-Coenzyme A binding domain containing 3 | 1.7 |
| PEX13 | peroxisome biogenesis factor 13 | 1.7 |
| AP3D1 | adaptor-related protein complex 3, delta 1 subunit | 1.6 |
| AP1S2 | adaptor-related protein complex 1, sigma 2 subunit | 1.5 |
| PEX1 | peroxisome biogenesis factor 1 | 1.5 |
| SNX1 | sorting nexin 1 | 1.5 |
| VPS45A | vacuolar protein sorting 45A (yeast) | 1.4 |
|  |  |  |
| **Miscellaneous** | |  |
| SERPINE1 | serine (or cysteine) proteinase inhibitor, clade E (nexin) | 5.6 |
| C5R1 | complement component 5 receptor 1 (C5a ligand) | 5.3 |
| FABP6 | fatty acid binding protein 6, ileal (gastrotropin) | 5.1 |
| IL21R | interleukin 21 receptor | 4.9 |
| CDK9 | cyclin-dependent kinase 9 (CDC2-related kinase) | 1.7 |
| SREBF1 | sterol regulatory element binding transcription factor 1 | 1.4 |
