# Supplementary material for: Characterization of two candidate genes, NCoA3 and IRF8, potentially involved in the control of HIV-1 latency
Source: Retrovirology. 2005 Nov 23;2:73. doi: 10.1186/1742-4690-2-73 (PMC1310520; doi:10.1186/1742-4690-2-73)
Supplement: Additional File 3 — Genes specifically upregulated in ACH-2 cells. [file 1742-4690-2-73-S3.doc]

| **Symbol** | **Name** | **ACH2NaBvsACH2 Signal log_2_ ratio** |
| --- | --- | --- |
|  |  |  |
| **Transcription** | |  |
| SIRT5 | sirtuin (silent mating type information regulation 2 homolog) 5 (S. cerevisiae) | 3.5 |
| IRF7 | interferon regulatory factor 7 | 2.5 |
| HEY1 | hairy/enhancer-of-split related with YRPW motif 1 | 1.9 |
| FOXO1A | forkhead box O1A (rhabdomyosarcoma) | 1.6 |
| SMAD7 | SMAD, mothers against DPP homolog 7 (Drosophila) | 1.4 |
| DLX4 | distal-less homeobox 4 | 1.3 |
| NFRKB | nuclear factor related to kappa B binding protein | 1.3 |
| PPP1R10 | protein phosphatase 1, regulatory subunit 10 | 1.3 |
| ZFP95 | zinc finger protein 95 homolog (mouse) | 1.3 |
| CEBPB | CCAAT/enhancer binding protein (C/EBP), beta | 1.2 |
|  |  |  |
| **Signal Transduction** | |  |
| PARG1 | PTPL1-associated RhoGAP 1 | 3.3 |
| NRGN | neurogranin (protein kinase C substrate, RC3) | 3 |
| CDC42EP3 | CDC42 effector protein (Rho GTPase binding) 3 | 2.5 |
| RAB3B | RAB3B, member RAS oncogene family | 2.1 |
| CAPN5 | calpain 5 | 1.6 |
| OPTN | optineurin | 1.6 |
| EPOR | erythropoietin receptor | 1.4 |
| PTPN1 | protein tyrosine phosphatase, non-receptor type 1 | 1.4 |
| IL6ST | Interleukin 6 signal transducer (gp130, oncostatin M receptor) | 1.3 |
| RALB | v-ral simian leukemia viral oncogene homolog B | 1.3 |
|  |  |  |
| **Protein Transport** | |  |
| BRDG1 | BCR downstream signaling 1 | 1.8 |
| STX7 | syntaxin 7 | 1.2 |
| VPS16 | vacuolar protein sorting 16 (yeast) | 1.2 |
| COPE | coatomer protein complex, subunit epsilon | 1 |
|  |  |  |
| **Metabolism** | |  |
| DHRS2 | dehydrogenase/reductase (SDR family) member 2 | 8.1 |
| FTCD | formiminotransferase cyclodeaminase | 6.4 |
| BG1 | lipidosin | 4.4 |
| ACSL1 | acyl-CoA synthetase long-chain family member 1 | 3.4 |
| ASNS | asparagine synthetase | 3.1 |
| ALDOC | aldolase C, fructose-bisphosphate | 3 |
| INSIG1 | insulin induced gene 1 | 2.8 |
| LSS | lanosterol synthase (2,3-oxidosqualene-lanosterol cyclase) | 2.4 |
| HMGCS1 | 3-hydroxy-3-methylglutaryl-Coenzyme A synthase 1 (soluble) | 2 |
| IDI1 | isopentenyl-diphosphate delta isomerase | 1.7 |
|  |  |  |
| **Miscellaneous** | |  |
| DHRS2 | dehydrogenase/reductase (SDR family) member 2 | 8.1 |
| HIST1H2BG | histone 1, H2bg | 8 |
| C1S | complement component 1, s subcomponent | 7.1 |
| RPL3L | ribosomal protein L3-like | 6.5 |
| FTCD | formiminotransferase cyclodeaminase | 6.4 |
| CYP4F3 | cytochrome P450, family 4, subfamily F, polypeptide 3 | 6.2 |
| RCN3 | reticulocalbin 3, EF-hand calcium binding domain | 5.2 |
| RIS1 | Ras-induced senescence 1 | 5.2 |
