# Supplementary material for: Characterization of two candidate genes, NCoA3 and IRF8, potentially involved in the control of HIV-1 latency
Source: Retrovirology. 2005 Nov 23;2:73. doi: 10.1186/1742-4690-2-73 (PMC1310520; doi:10.1186/1742-4690-2-73)
Supplement: Additional File 4 — Genes downregulated in U1 and ACH-2 cells. [file 1742-4690-2-73-S4.doc]

| **Symbol** | **Name** | **U1NaBvsU1 Signal log_2_ ratio** | **ACH2NaBvsACH2 Signal log_2_ ratio** |
| --- | --- | --- | --- |
|  |  |  |  |
| **Transcription** | |  |  |
| FLI1 | Friend leukemia virus integration 1 | -7.8 | -2.4 |
| MYC | v-myc myelocytomatosis viral oncogene homolog (avian) | -4.8 | -4.8 |
| NMI | N-myc (and STAT) interactor | -4.2 | -4.3 |
| MYB | v-myb myeloblastosis viral oncogene homolog (avian) | -3.8 | -2.8 |
| TOE1 | target of EGR1, member 1 (nuclear) | -3.7 | -1.6 |
| ZNF278 | zinc finger protein 278 | -3.6 | -2.4 |
| CHD3 | chromodomain helicase DNA binding protein 3 | -3 | -3.4 |
| SATB1 | special AT-rich sequence binding protein 1 | -3 | -2.9 |
| PHF11 | PHD finger protein 11 | -3 | -1.8 |
| PRKCBP1 | protein kinase C binding protein 1 | -2.7 | -4.3 |
| IRAK1 | interleukin-1 receptor-associated kinase 1 | -2.7 | -2.7 |
|  |  |  |  |
| **Signal Transduction** | |  |  |
| IL27RA | interleukin 27 receptor, alpha | -4.5 | -3.1 |
| ADRBK1 | adrenergic, beta, receptor kinase 1 | -3.8 | -2.9 |
| TRAF3 | TNF receptor-associated factor 3 | -3 | -1.3 |
| ARHGAP4 | Rho GTPase activating protein 4 | -2.9 | -2.4 |
| ITPKB | inositol 1,4,5-trisphosphate 3-kinase B | -2.8 | -2 |
| IRAK1 | interleukin-1 receptor-associated kinase 1 | -2.7 | -2.7 |
| LRP8 | low density lipoprotein receptor-related protein 8 | -2.5 | -1.7 |
| IL2RG | interleukin 2 receptor, gamma | -2.4 | -2.6 |
| PECAM1 | platelet/endothelial cell adhesion molecule (CD31 antigen) | -2.4 | -2.1 |
| HDGF | hepatoma-derived growth factor (high-mobility group protein-like) | -2.3 | -1.9 |
| EVL | Enah/Vasp-like | -2.3 | -1.7 |
| PTPRC | protein tyrosine phosphatase, receptor type, C | -2.2 | -3.4 |
|  |  |  |  |
| **Immune Response** | |  |  |
| LCP2 | lymphocyte cytosolic protein 2 | -6.9 | -3.2 |
| PSMB8 | proteasome (prosome, macropain) subunit, beta type, 8 | -2.8 | -3.4 |
| PSMB10 | proteasome (prosome, macropain) subunit, beta type, 10 | -2 | -2.9 |
| TAPBP | TAP binding protein (tapasin) | -2 | -1.8 |
| CTSC | cathepsin C | -1.9 | -2.1 |
| IFITM2 | interferon induced transmembrane protein 2 (1-8D) | -1.9 | -1.4 |
|  |  |  |  |
| **RNA Modification** | |  |  |
| QTRT1 | queuine tRNA-ribosyltransferase 1 | -4.1 | -2.7 |
| CUGBP2 | CUG triplet repeat, RNA binding protein 2 | -3.7 | -2.2 |
| PUS1 | pseudouridylate synthase 1 | -2.5 | -2 |
| HNRPUL1 | heterogeneous nuclear ribonucleoprotein U-like 1 | -2.4 | -1.6 |
| HNRPH3 | heterogeneous nuclear ribonucleoprotein H3 (2H9) | -2.3 | -2.2 |
| FLJ20244 | hypothetical protein FLJ20244 | -2.1 | -2.8 |
|  |  |  |  |
| **Miscellaneous** | |  |  |
| MLSTD1 | male sterility domain containing 1 | -5.8 | -2.8 |
| LRMP | lymphoid-restricted membrane protein | -5.7 | -2.1 |
| BDH | 3-hydroxybutyrate dehydrogenase (heart, mitochondrial) | -5.3 | -2.6 |
| DOCK10 | dedicator of cytokinesis 10 | -4.7 | -2.1 |
| LOC150759 | hypothetical protein LOC150759 | -4.5 | -2 |
| CAS1 | O-acetyltransferase | -4.4 | -2.2 |
| FMNL1 | formin-like 1 | -4.2 | -4.2 |
