# Supplementary material for: Characterization of two candidate genes, NCoA3 and IRF8, potentially involved in the control of HIV-1 latency
Source: Retrovirology. 2005 Nov 23;2:73. doi: 10.1186/1742-4690-2-73 (PMC1310520; doi:10.1186/1742-4690-2-73)
Supplement: Additional File 5 — Genes specifically downregulated in U1 cells. [file 1742-4690-2-73-S5.doc]

| **Symbol** | **Name** | **U1NaBvsU1 Signal log_2_ ratio** |
| --- | --- | --- |
|  |  |  |
| **Transcription** | |  |
| SPI1 | spleen focus forming virus (SFFV) proviral integration oncogene spi1 | -5.3 |
| ZFHX1B | zinc finger homeobox 1b | -4.6 |
| ELF4 | E74-like factor 4 (ets domain transcription factor) | -4.3 |
| HLF | hepatic leukemia factor | -4.2 |
| POU2F2 | POU domain, class 2, transcription factor 2 | -4.1 |
| **IRF8** | **interferon regulatory factor 8** | **-4** |
| GFI1 | growth factor independent 1 | -3.6 |
| NFE2 | nuclear factor (erythroid-derived 2), 45kDa | -3 |
| MNDA | myeloid cell nuclear differentiation antigen | -2.7 |
| MAFG | v-maf musculoaponeurotic fibrosarcoma oncogene homolog G (avian) | -2.6 |
| SHOX2 | short stature homeobox 2 | -2.6 |
| STAT5A | signal transducer and activator of transcription 5A | -2.6 |
|  |  |  |
| **Signal Transduction** | |  |
| RAB33A | RAB33A, member RAS oncogene family | -3.7 |
| CIAS1 | cold autoinflammatory syndrome 1 | -3.4 |
| CLECSF5 | C-type (calcium dependent) lectin, superfamily member 5 | -3.4 |
| NSMAF | neutral sphingomyelinase (N-SMase) activation associated factor | -3.3 |
| IL18RAP | interleukin 18 receptor accessory protein | -3.1 |
| CIDEB | cell death-inducing DFFA-like effector b | -2.9 |
| PYCARD | PYD and CARD domain containing | -2.8 |
| RGS19 | regulator of G-protein signalling 19 | -2.5 |
| TLR2 | toll-like receptor 2 | -2.5 |
| IL4R | interleukin 4 receptor | -2.4 |
|  |  |  |
| **Immune Response** | |  |
| EBI2 | Epstein-Barr virus induced gene 2 | -5.5 |
| FCAR | Fc fragment of IgA, receptor for | -4.1 |
| IL18RAP | interleukin 18 receptor accessory protein | -3.1 |
| HLA-DOB | major histocompatibility complex, class II, DO beta | -3 |
| LILRA2 | leukocyte immunoglobulin-like receptor, subfamily A, member 2 | -2.9 |
| LY64 | lymphocyte antigen 64 homolog, radioprotective 105kDa (mouse) | -2.6 |
| CST7 | cystatin F (leukocystatin) | -2.5 |
| FCGR1A | Fc fragment of IgG, high affinity Ia, receptor for (CD64) | -2.3 |
| MX2 | myxovirus (influenza virus) resistance 2 (mouse) | -2.1 |
|  |  |  |
| **RNA Modification** | |  |
| NXF3 | nuclear RNA export factor 3 | -3 |
| RPP40 | ribonuclease P 40kDa subunit | -1.7 |
| SEN2L | likely homolog of yeast SEN2 | -1.6 |
| FLJ20485 | hypothetical protein FLJ20485 | -1.4 |
| RBM5 | RNA binding motif protein 5 | -1.4 |
| DDX18 | DEAD (Asp-Glu-Ala-Asp) box polypeptide 18 | -1.1 |
|  |  |  |
| **Miscellaneous** | |  |
| TARP | TCR gamma alternate reading frame protein | -6.9 |
| LOC146712 | hypothetical protein LOC146712 | -6.6 |
| FLJ22457 | hypothetical protein FLJ22457 | -5.5 |
| FES | feline sarcoma oncogene | -5.4 |
| MFAP4 | microfibrillar-associated protein 4 | -5.2 |
| TFEC | transcription factor EC | -5 |
