# Supplementary material for: Characterization of two candidate genes, NCoA3 and IRF8, potentially involved in the control of HIV-1 latency
Source: Retrovirology. 2005 Nov 23;2:73. doi: 10.1186/1742-4690-2-73 (PMC1310520; doi:10.1186/1742-4690-2-73)
Supplement: Additional File 6 — Genes specifically downregulated in ACH-2 cells. [file 1742-4690-2-73-S6.doc]

| **Symbol** | **Name** | **ACH2NaBvsACH2 Signal log_2_ ratio** |
| --- | --- | --- |
|  |  |  |
| **Transcription** | |  |
| WT1 | Wilms tumor 1 | -4.6 |
| GABPB2 | GA binding protein transcription factor, beta subunit 2, 47kDa | -4.1 |
| ZHX2 | zinc fingers and homeoboxes 2 | -3.4 |
| ERG | v-ets erythroblastosis virus E26 oncogene like (avian) | -3.1 |
| NKX2-5 | NK2 transcription factor related, locus 5 (Drosophila) | -3.1 |
| IFI16 | interferon, gamma-inducible protein 16 | -3 |
| MBD3 | methyl-CpG binding domain protein 3 | -3 |
| LEF1 | lymphoid enhancer-binding factor 1 | -2.8 |
| UBTF | upstream binding transcription factor, RNA polymerase I | -2.8 |
| MYCN | v-myc myelocytomatosis viral related oncogene, neuroblastoma derived | -2.7 |
| RHOH | ras homolog gene family, member H | -2.7 |
| GTF2I | general transcription factor II, i | -2.6 |
|  |  |  |
| **Signal Transduction** | |  |
| TRIM | T-cell receptor interacting molecule | -6.5 |
| TP53 | tumor protein p53 (Li-Fraumeni syndrome) | -3.1 |
| LCK | lymphocyte-specific protein tyrosine kinase | -2.8 |
| SIT | SHP2-interacting transmembrane adaptor protein | -2.8 |
| GTF2I | general transcription factor II, i | -2.6 |
| CD3Z | CD3Z antigen, zeta polypeptide (TiT3 complex) | -2.4 |
| VAV3 | vav 3 oncogene | -2.4 |
| CD3G | CD3G antigen, gamma polypeptide (TiT3 complex) | -2.2 |
| SPRY1 | sprouty homolog 1, antagonist of FGF signaling (Drosophila) | -2.1 |
| TNFRSF6 | tumor necrosis factor receptor superfamily, member 6 | -2 |
| TRAF5 | TNF receptor-associated factor 5 | -1.7 |
|  |  |  |
| **Immune Response** | |  |
| ARTS-1 | type 1 tumor necrosis factor receptor shedding aminopeptidase regulator | -5.3 |
| IGLL1 | immunoglobulin lambda-like polypeptide 1 | -4.3 |
| ZAP70 | zeta-chain (TCR) associated protein kinase 70kDa | -2.8 |
| GZMA | granzyme A (cytotoxic T-lymphocyte-associated serine esterase 3) | -2.5 |
| LOC91316 | similar to bK246H3.1 (immunoglobulin lambda-like polypeptide 1) | -2.3 |
| TRB | T cell receptor beta locus | -2.3 |
| NOTCH1 | Notch homolog 1, translocation-associated (Drosophila) | -2 |
| IFITM1 | interferon induced transmembrane protein 1 (9-27) | -1.6 |
|  |  |  |
| **RNA Modification** | |  |
| IMP-3 | IGF-II mRNA-binding protein 3 | -1.9 |
| TARBP1 | TAR (HIV) RNA binding protein 1 | -1.2 |
| FMR1 | fragile X mental retardation 1 | -1.1 |
| HNRPR | heterogeneous nuclear ribonucleoprotein R | -1.1 |
| HNRPU | heterogeneous nuclear ribonucleoprotein U (scaffold attachment factor A) | -1.1 |
| DDX39 | DEAD (Asp-Glu-Ala-Asp) box polypeptide 39 | -1.2 |
|  |  |  |
| **Miscellaneous** | |  |
| LRAP | leukocyte-derived arginine aminopeptidase | -5.2 |
| SH2D1A | SH2 domain protein 1A, Duncan's disease (lymphoproliferative syndrome) | -5.1 |
| STAG3 | stromal antigen 3 | -5 |
| LMO2 | LIM domain only 2 (rhombotin-like 1) | -4.8 |
| MICAL-L1 | MICAL-like 1 | -4.6 |
